# Altered weight-bearing compensatory neuromuscular control strategies in early-stage hip-related pain

**DOI:** 10.3389/fspor.2026.1810862

**Published:** 2026-05-25

**Authors:** Qiyuan Lin, Muchen Ren, Jian Jin, Jingzhou Wang, Ji Hou, Qing Sun, Bo Huo, Jianhao Lin

**Affiliations:** 1 Arthritis Clinical and Research Center, Peking University People’s Hospital, Beijing, China; 2 Arthritis Institute, Peking University, Beijing, China; 3 Department of Rehabilitation, Peking University People’s Hospital, Beijing, China; 4 Institute of Artificial Intelligence in Sports, Capital University of Physical Education and Sports, Beijing, China

**Keywords:** compensatory neuromuscular control strategies, hip-related pain, muscle synergies, squat, surface electromyography

## Abstract

**Introduction:**

Individuals with early hip–related pain may exhibit subtle compensatory neuromuscular alterations even when overall movement performance appears largely preserved, making these changes difficult to detect through routine observation. Whether modulation of stance width during a weight–bearing task can provoke and reveal these underlying control differences remains unclear.

**Methods:**

Participants with early hip–related pain and asymptomatic controls performed standardized squats under three stance–width conditions. Bilateral surface electromyography signals were recorded from the lower limbs during task execution. Time-varying electromyography and muscle synergy analyses were used to compare neuromuscular control characteristics between groups.

**Results:**

Both groups demonstrated three stable muscle synergy patterns across conditions. However, the hip pain group showed specific adaptations in synergy weights and activation timing, with group differences mainly observed during the squat–bottom holding phase and the transition to ascent. These differences became more pronounced as stance width increased. In addition, the hip pain group showed reduced maximal voluntary contraction strength, whereas electromyographic frequency–domain features remained relatively stable

**Discussion:**

Early hip–related pain appears to be characterized more by task–dependent compensatory reorganization than by overt functional deficits. Modulation of stance width can effectively provoke aberrant movement patterns and, when combined with electromyographic monitoring, may serve as a useful research tool for identifying underlying neuromuscular control differences.

## Introduction

Hip-related pain is a common condition in sports medicine and orthopaedic practice, characterized by complex etiology and substantial clinical heterogeneity, which extends beyond diagnostic categories and structural findings to include variability in functional performance and motor control strategies (1–3). In the earliest stages of the condition, clinical symptoms and imaging abnormalities are often mild or absent; however, hip-related pain is commonly accompanied by varying degrees of muscle weakness and muscle atrophy (4, 5), and the absence of overt clinical signs does not necessarily indicate preserved neuromuscular function. Accumulating evidence indicates that individuals may already exhibit subtle functional compensations and alterations in motor control during daily weight-bearing activities, even when overall task performance appears acceptable (6, 7). Although such compensatory strategies may temporarily preserve movement outcomes, failure to identify and address them in a timely manner may result in chronic overload of non-symptomatic regions and maladaptive redistribution of mechanical and neuromuscular demands across periarticular tissues, thereby increasing the risk of secondary injury and symptom progression (8, 9). Consequently, accurately identifying early movement and neuromuscular characteristics in individuals with hip-related pain, particularly at the level of coordination strategies rather than overt deficits, prior to the emergence of pronounced clinical signs, is critical for early assessment and targeted intervention.

Squatting is a functional movement with high clinical relevance and can serve as a valuable task for identifying underlying neuromuscular deficiencies in individuals with hip-related pain (10, 11). As a typical closed-chain, weight-bearing task, squatting requires substantial hip flexion and coordinated control across multiple muscles and joints, placing high demands not only on force production but also on intermuscular coordination and postural stability (12–14). Notably, because gross kinematics are often preserved on a subjective scale in patients compared to healthy individuals, the bilateral squat provides a unique platform to elicit latent compensatory muscle activation strategies that may not be apparent through visual observation alone. Consequently, while the task itself is easy to administer, discerning these subtle neuromuscular control deficits requires a more granular analysis of activation patterns, particularly under conditions that constrain available movement solutions.

To further elucidate the motor control mechanisms associated with hip-related pain, reliance on kinematic measures alone is often insufficient to capture underlying neuromuscular regulation, especially in early-stage conditions where joint motion patterns may appear largely normal. Electromyographic analysis allows direct quantification of muscle activation magnitude and timing during functional tasks and provides important insight into load tolerance and neuromuscular recruitment (15). However, metrics derived from single muscles or activation amplitude alone are limited in their ability to represent coordinated activity across multiple muscles during complex movements and therefore provide limited explanatory power regarding compensatory neuromuscular control strategies. Muscle synergy analysis addresses this limitation by offering a framework to examine muscle control at the level of coordination strategies, characterizing how multiple muscles are organized in a modular manner to accomplish task demands. This approach enables the identification of strategy-level reorganization that may occur without obvious changes in overall movement patterns or performance (16–18), thereby facilitating the detection of subtle alterations in neuromuscular control. Accordingly, integrating conventional EMG features with muscle synergy analysis enables a more comprehensive characterization of neuromuscular control in individuals with hip-related pain, capturing both activation magnitude and coordination structure.

Accordingly, this study compared neuromuscular control during squatting between individuals with early-stage or non-surgical chronic hip pain and asymptomatic adults. Stance width was manipulated to progressively increase stability demands, thereby systematically constraining motor solutions and amplifying coordination requirements, and neuromuscular control was characterized using a combination of EMG-derived measures and muscle synergy analysis. The aims were to determine under which stance conditions between-group differences are most readily detected as task constraints increase, and to examine the extent to which these differences manifest as alterations in coordination strategies, alongside changes in neuromuscular activation and cost. By focusing on task-dependent and phase-specific control features, this study seeks to clarify how early hip-related pain is associated with compensatory neuromuscular strategies that may precede overt clinical deterioration, and to support the use of clinically actionable task parameters for assessment and rehabilitation progression.

## Methods

### Participants

This study recruited individuals with chronic hip pain and age-similar asymptomatic adults from the community. The hip-pain group included 21 participants (9 males, 42.9%), and the control group included 10 participants (5 males, 50.0%) (Table 1). No significant differences were observed between the two groups in terms of age, height, weight, or body mass index. Participants in the hip pain group reported experiencing recurrent hip pain for at least 6 months, with no substantial changes in pain intensity or character over the past month. All participants in this group were clinically diagnosed with femoroacetabular impingement syndrome, and pain could be provoked by specific physical examination tests, such as the flexion-adduction-internal rotation (FADIR) test and flexion-abduction-external rotation (FABER) test (1, 19). Additionally, radiographic imaging confirmed the presence of cam or pincer morphologies, characteristic of femoroacetabular impingement. To ensure diagnostic consistency, each participant underwent a comprehensive physical examination and imaging evaluation, confirming the diagnosis of femoroacetabular impingement.

**Table 1:** Participant characteristics.

Characteristic	Pain Group (*n* = 21)	Control Group (*n* = 10)	*p*-value
Age (years)	36.33 ± 6.58	37.70 ± 3.41	0.9932
Height (cm)	170.61 ± 7.49	170.90 ± 10.45	>0.9999
Body mass (kg)	66.23 ± 13.89	69.50 ± 16.54	0.8530
BMI (kg/m^2^)	22.57 ± 3.39	23.45 ± 3.41	0.9988
Males (*n*, %)	9 (42.85%)	5 (50%)	

Exclusion criteria for both groups included: inflammatory arthritis or other significant musculoskeletal disorders; a history of substantial lower limb trauma or surgery; rehabilitation therapy or intra-articular injections within the past 6 months; the use of assistive devices; the use of analgesics that could affect neuromuscular function within the past 2 weeks (e.g., nonsteroidal anti-inflammatory drugs); relevant neurological or metabolic conditions such as diabetes, thyroid disorders, or peripheral neuropathy; inability to complete the tasks due to severe functional limitations or cardiopulmonary disease; and a BMI greater than 32 kg/m^2^.

All participants provided written informed consent prior to participation. The experimental protocol was approved by the Institutional Review Board of Peking University People's Hospital (Approval No. 2024PHB575-001), and all procedures were conducted in accordance with the Declaration of Helsinki.

### Procedures

Surface electromyography EMG was recorded bilaterally using 16 wireless sensors from the Trigno system by Delsys Inc. (Natick, Massachusetts, USA), at a sampling rate of 2,000 Hz and time-synchronized with kinematic and kinetic measurements. Following the SENIAM recommendations, electrodes were placed on both legs over the gluteus maximus, gluteus medius, rectus femoris, vastus medialis, vastus lateralis, tensor fasciae latae, semitendinosus, and biceps femoris. Before electrode placement, participants completed a 5-min fast walk on a treadmill for warm-up. The skin was prepared by shaving when needed and cleaning with 75% alcohol to reduce impedance. Prior to data collection, brief isometric contractions of the target muscles were performed to verify signal quality.

Participants performed squat trials under three stance-width conditions (40 cm, 60 cm, and 80 cm), with the order of conditions randomly assigned to each participant to control for potential order effects. Each trial began from an upright standing position, with EMG recording starting as participants prepared to initiate the squat. Participants were instructed to perform a slow, controlled descent for approximately 2 s, hold the bottom position for 5 s, and then return to an upright standing posture with a slow, controlled ascent, also lasting about 2 s. Sufficient rest was provided between trials to ensure full recovery. In the hip-pain group, participants were encouraged to squat to a depth that was comfortable and did not provoke pain, but squat depth was not strictly enforced. At least three trials were completed for each stance-width condition.

**Scheme 1:**
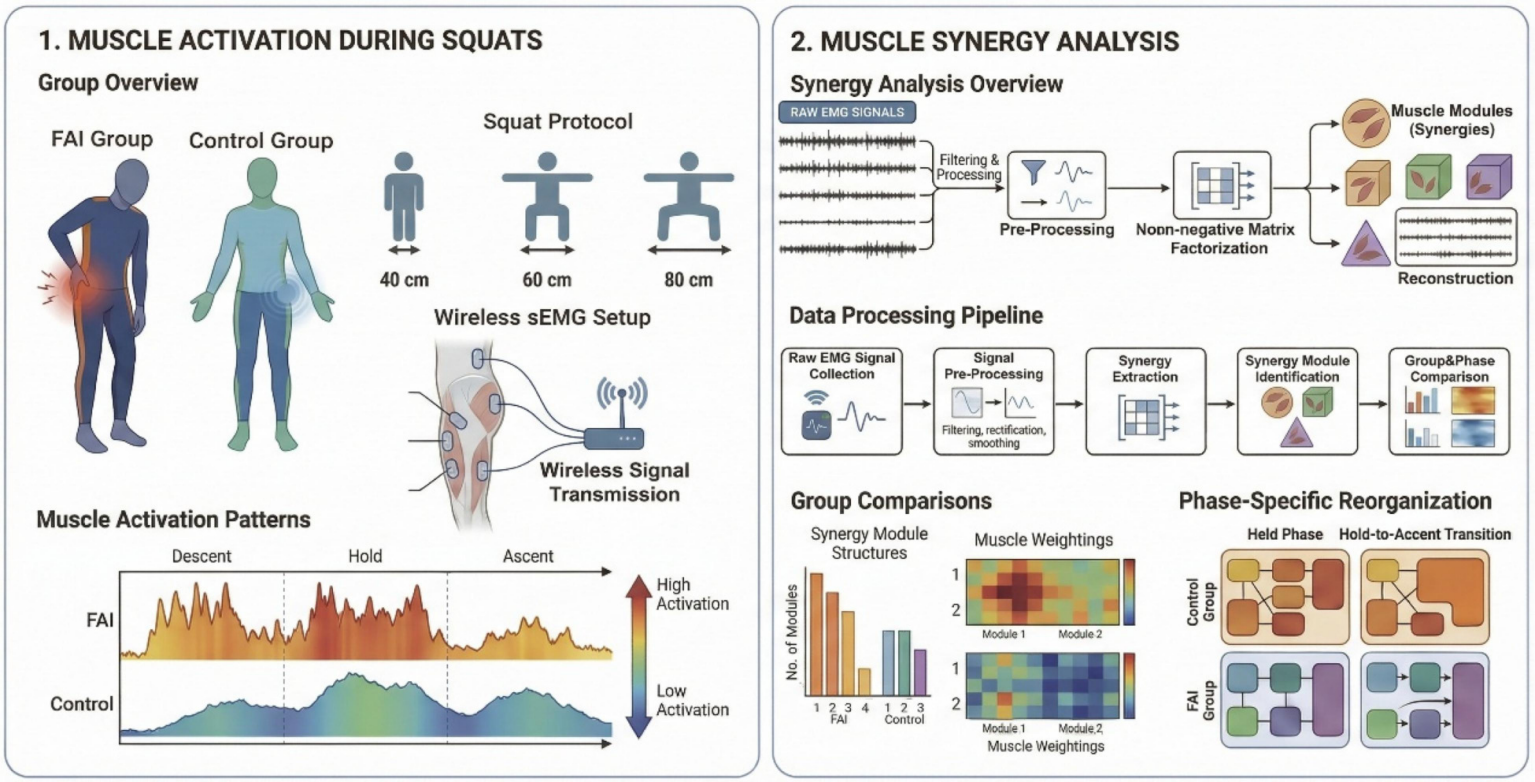
Schematic diagram of the experimental protocol and data analysis pipeline.

The onset and offset of the EMG data were synchronized with the kinematic and kinetic measurements using the motion capture system (Prime 13, Optitrack, US). The squat cycle was clearly defined as follows: the cycle began when the participant initiated the descent, which was determined by the first detectable knee flexion, defined as a knee angle of 10° as measured by the motion capture system. The cycle ended when the participant returned to an upright posture, with the knee angle at 0° and the trunk aligned vertically. This definition ensured consistent event detection across participants and minimized artificial temporal variability. EMG data were time-stamped based on the effective movement time recorded by the motion capture system, allowing for accurate measurement of muscle activation during the squat (Scheme 1).

### Maximum voluntary isometric contraction (MVIC) protocol

Following SENIAM and Kendall's guidelines, MVIC tests were performed for sEMG normalization. Participants completed three 5-second maximal contractions for each muscle with 60-second rest intervals, while receiving standardized verbal encouragement and manual stabilization. Testing positions were strictly standardized for each muscle: (1) Rectus Femoris (RF), Vastus Medialis (VM), and Vastus Lateralis (VL): seated with the hip and knee flexed at 90°, with resistance applied against knee extension; (2) Gluteus Maximus (Gmax): prone with the knee flexed at 90°, with resistance against hip extension; (3) Gluteus Medius (Gmed): side-lying, with resistance against hip abduction; (4) Biceps Femoris (BF) and Semitendinosus (ST): prone with the knee flexed to 45°, with the lower leg externally rotated for BF or internally rotated for ST, and resistance applied against knee flexion; (5) Tensor Fasciae Latae (TFL): supine with the hip flexed to 30°, abducted, and internally rotated, with resistance applied against combined hip flexion and abduction.

### EMG preprocessing and normalization

In the general FAI patient population, even when symptoms are initially present in only one hip, the contralateral hip often exhibits similar bony abnormalities (20–22). Therefore, we analyzed the bilateral data from 16 muscles in both the hip pain and control groups to gain a comprehensive understanding of potential compensatory mechanisms and structural abnormalities in both hips. Raw surface EMG signals were band-pass filtered using a fourth-order Butterworth filter (20–500 Hz) and notch filtered at 50 Hz to remove power-line interference. The filtered signals were full-wave rectified. For EMG waveform analysis, a 100-ms moving RMS window was applied to generate the EMG amplitude envelope.

For normalization, muscle-specific MVIC tests were performed using standardized isometric test positions for each muscle. EMG data from the MVIC trials were processed using the same filtering, rectification, and 100 ms RMS procedures as the squat trials. For each muscle, the highest peak RMS value obtained across valid MVIC trials was used as the normalization reference. Squat EMG signals were then normalized to the corresponding MVIC reference and expressed as %MVIC.

### Time-normalized EMG waveform analysis

**Figure 1:**
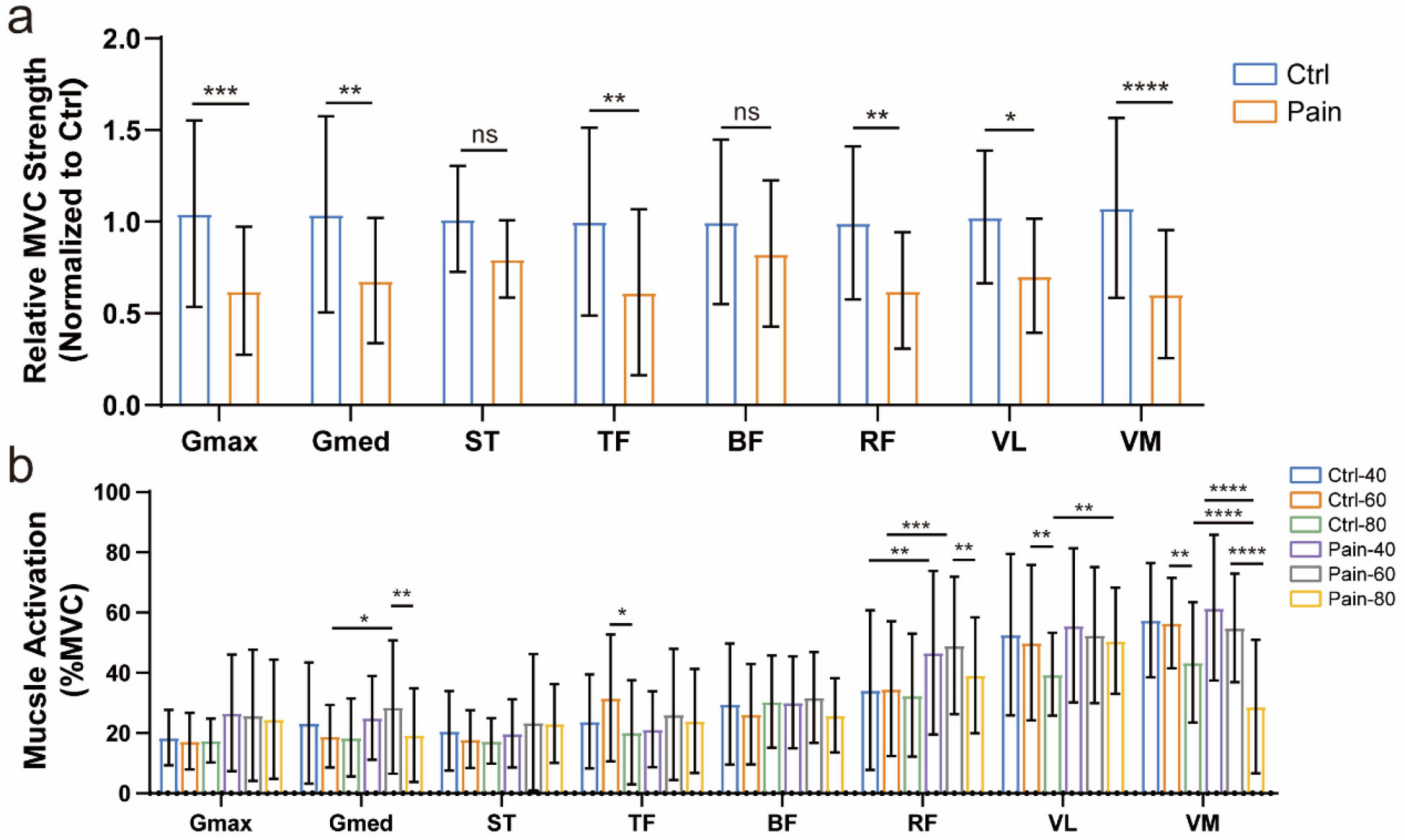
MVIC-normalized EMG activation profiles of eight muscles during squats performed with different stance widths. **(a)** Group-averaged EMG activation profiles of each muscle during squats performed with a 40-cm inter-foot distance. **(b)** Group-averaged EMG activation profiles of each muscle during squats performed with a 60-cm inter-foot distance. **(c)** Group-averaged EMG activation profiles of each muscle during squats performed with an 80-cm inter-foot distance. EMG amplitudes were normalized to MVIC and plotted across the full squat cycle. The control group is shown in blue, and the hip-related pain group is shown in orange. Thick lines represent group means, and shaded regions indicate ±1 SD.

Each valid squat trial was segmented into one complete squat cycle and time-normalized to 0%–100% of the movement cycle. The waveform was resampled to 101 data points using linear interpolation. For each muscle, the RMS EMG value at each time point was divided by the corresponding MVIC reference and multiplied by 100, yielding a time-varying activation profile expressed as %MVIC. These normalized waveforms were used to generate the EMG activation profiles shown in Figure 1.

Between-group differences in EMG waveforms were evaluated using one-dimensional non-parametric statistical parametric mapping (SnPM) within each stance-width condition. The full width at half maximum (FWHM) was estimated for each condition to characterize the smoothness of the residual field.

### EMG amplitude variables

To further quantify muscle activation magnitude during squatting, peak EMG and iEMG were calculated for each muscle in each trial. Peak EMG was defined as the maximum value of the MVIC-normalized RMS waveform during the squat cycle and was expressed as %MVIC. iEMG was defined as the time integral of the normalized, full-wave rectified EMG signal over the squat cycle, representing cumulative muscle activation during the movement. For each participant and condition, values were averaged across valid trials prior to statistical analysis.

### Muscle synergy analysis

Muscle synergies were extracted using non-negative matrix factorization (NNMF). For each trial, the time-normalized EMG data were organized into an 8 × 101 matrix, corresponding to eight muscles and 101 time points. When three valid trials were available, the matrices were concatenated along the time dimension to form an 8 × 303 matrix. NNMF decomposed the EMG matrix into a muscle-weighting matrix (W) and an activation-timing matrix (H), such that the original EMG matrix was approximated by WH. Reconstruction quality was assessed using variance accounted for (VAF).

The number of modules was determined individually by increasing the number of synergies from 1 upward until VAF exceeded 90%; an additional module was retained only if it improved VAF by more than 5%. For group-level decomposition, EMG matrices from all participants within the same group were concatenated, and the number of modules was set as the maximum of the individual minimum module numbers. To assess between-group similarity, cross-reconstruction was performed by fixing the group-derived W matrix and re-estimating H for each participant's EMG data. Total and muscle-specific VAF values were calculated, and the decrease in VAF relative to the participant-specific decomposition was defined as *Δ*VAF. Larger *Δ*VAF values indicated lower similarity between the participant's EMG structure and the fixed group-derived synergy pattern.

### Statistical analysis

All continuous variables are presented as mean ± standard deviation. Normality was assessed using the Shapiro–Wilk test. Between-group comparisons were performed using an independent-samples *t*-test for normally distributed data and the Mann–Whitney *U*-test for non-normally distributed data. Scalar EMG-derived outcomes, including peak EMG, iEMG, RMS, %MVC and cross-reconstruction ΔVAF, were compared between the control and hip-pain groups separately under each stance-width condition of 40, 60, and 80 cm. Time-series data, including joint angles, joint moments, time-normalized EMG waveforms, and module activation profiles H, were compared between groups within each stance width using one-dimensional non-parametric statistical parametric mapping SnPM with permutation-based inference and thresholding across the full time domain. Correspondence between muscle-weighting vectors W was described using Pearson correlation coefficients and visualized as heatmaps. Statistical analyses other than SnPM were conducted in R version 4.2.2, and SnPM analyses were performed in Python 3.11 using spm1d version 0.4.18. Statistical significance was set at *p* < 0.05.

## Results

### EMG characteristics in the amplitude and frequency domains during squatting

**Figure 2:**
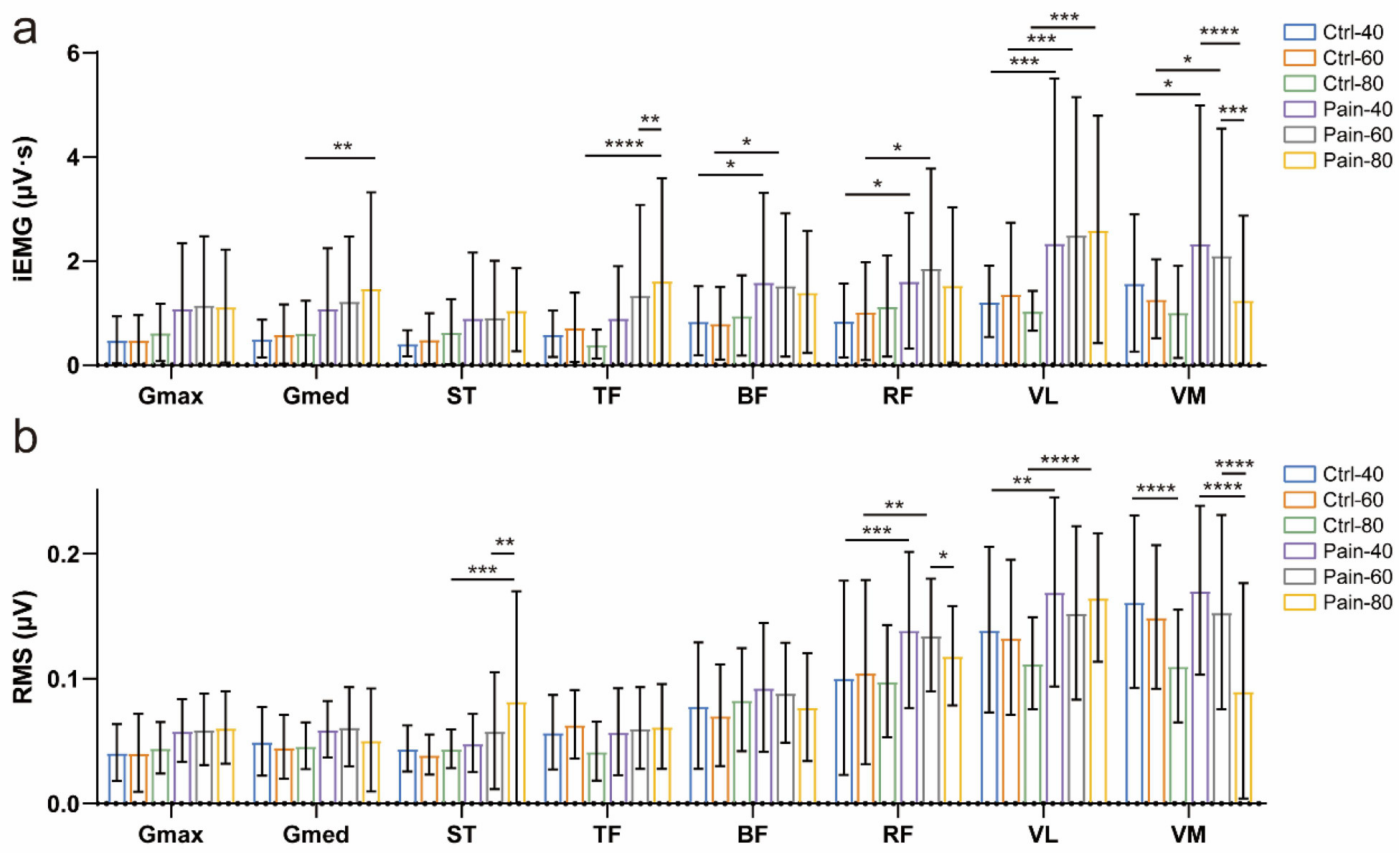
Maximal voluntary contraction and task-related muscle activation across stance widths. **(a)** Relative maximal voluntary contraction (MVC) strength of eight muscles in the control and hip-related pain groups, with MVC values normalized to the control-group mean for each muscle. **(b)** Muscle activation during squat trials expressed as %MVC across three stance widths with inter-foot distances of 40, 60, and 80 cm for both groups. Data are presented as mean ± s.d. Panel (a) was analyzed using independent-samples *t-*tests, and panel (b) was performed using one-way ANOVA followed by Tukey's *post hoc* test. Significance was indicated by asterisks: *P* < 0.05(*), *P* < 0.01(**), *P* < 0.001(***), and *P* < 0.0001 (****), ns, not significant.

Before the test movement began, the maximum voluntary isometric contraction (MVIC) signal was first measured for each participant following standardized procedures. We then analyzed the MVIC data for each muscle and compared it as a proportion of the control group's mean peak amplitude for the same muscle (Figure 2a). Overall, the MVIC of most muscles in the hip pain group was relatively lower, suggesting a decrease in the activation capacity of several hip and knee-related muscle groups. EMG amplitudes during squatting were then normalized to MVC to obtain relative activation levels (%MVC) for each stance width (Figure 2b). In general, %MVC values for the quadriceps muscles varied more markedly across stance widths and between groups and showed multiple significant differences, whereas %MVC differences for the gluteal muscles and parts of the hamstrings were fewer or less robust. This pattern was consistent with the iEMG and RMS findings, suggesting that between-group differences were more pronounced in knee extensor–related muscles and that stance width influenced relative recruitment for selected muscles.

**Figure 3:**
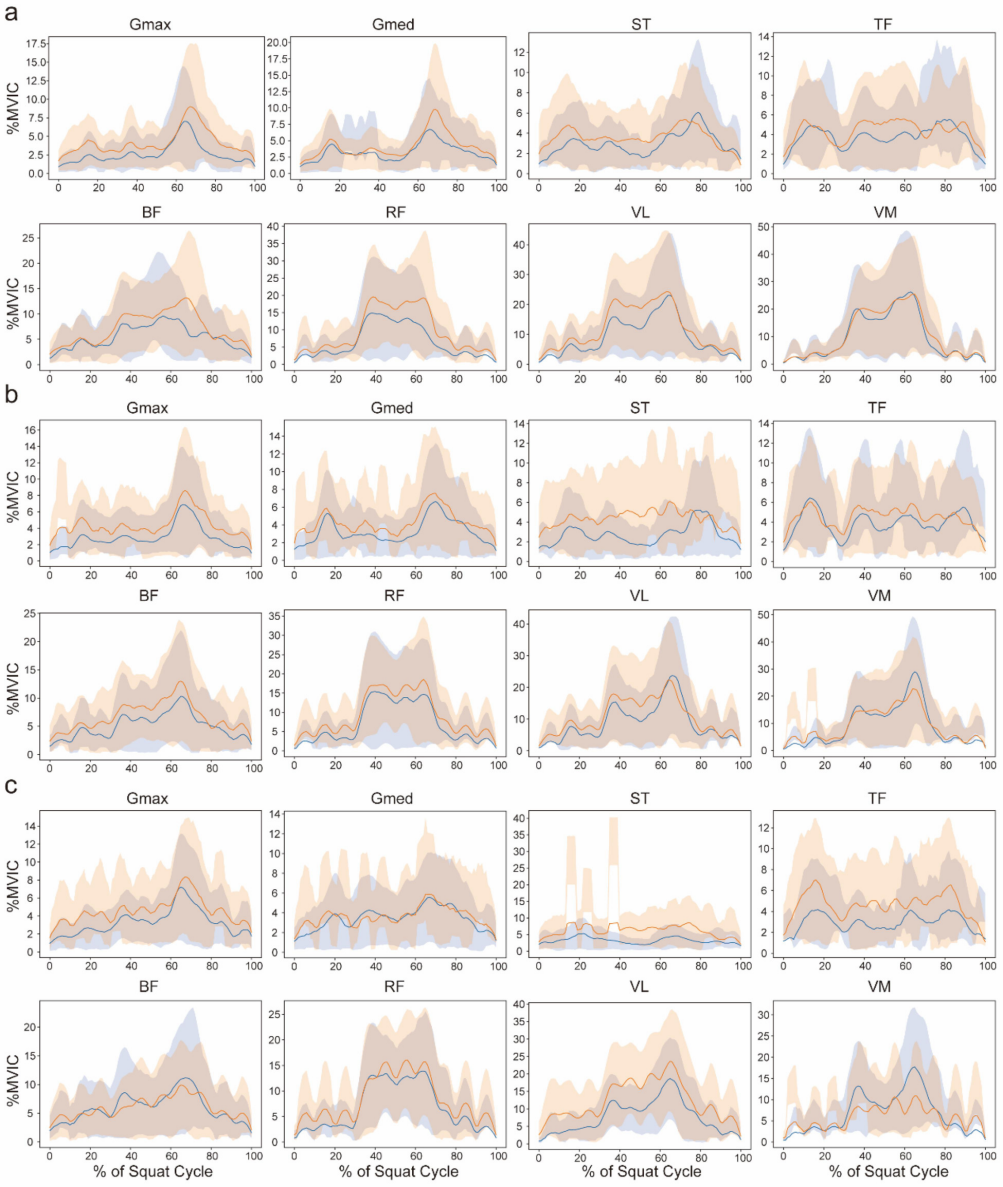
iEMG and RMS of eight muscles during squats performed with different stance widths. **(a)** Comparisons of iEMG among the three stance widths for each muscle under the control and pain conditions. **(b)** Comparisons of RMS among the three stance widths for each muscle under the control and pain conditions. Data are presented as mean ± s.d. Statistical comparisons were performed using one-way ANOVA followed by Tukey's *post hoc* test. Significance was indicated by asterisks: *P* < 0.05(*), *P* < 0.01(**), *P* < 0.001(***), and *P* < 0.0001 (****), ns, not significant.

Following the EMG waveform analysis, the activation amplitude characteristics of the eight muscles during squatting were further compared across different stance widths. One-way ANOVA with *post hoc* multiple comparisons revealed significant differences in the integrated EMG of several muscles, mainly including the Gmed, TF, BF, RF, VL, and VM, whereas the Gmax and ST showed smaller differences or no statistically significant effects. In terms of overall amplitude, the hip-related pain group generally exhibited higher integrated EMG levels in most muscles than the control group (Figure 3a). In addition, as stance width increased from 40 cm to 80 cm, the integrated EMG of several muscles in both groups showed an upward trend, although the within-group increases in some muscles did not reach statistical significance. Notably, the VM demonstrated an opposite pattern, with integrated EMG decreasing as stance width increased. Compared with integrated EMG, the significant effects observed in RMS were more limited, with significant between-group differences found only in the ST, RF, VL, and VM. In contrast, the significance of findings for the Gmed, TF, and BF was less consistent in RMS than in integrated EMG (Figure 3). This discrepancy may be related to the distinct physiological emphasis of the two indices: integrated EMG reflects the cumulative level of muscle activation over the movement cycle, whereas RMS is more sensitive to instantaneous amplitude, peak activity, and plateau-phase characteristics. Therefore, RMS may be more likely to detect statistically significant effects in muscles with greater amplitude fluctuations, such as the quadriceps and semitendinosus.

Figure 1 illustrates the MVIC-normalized EMG activation profiles of eight muscles across the time-normalized squat cycle under three stance-width conditions. The squat cycle was normalized to 0%–100%, with 0%–22% representing the descent phase, 22%–78% the holding phase, and 78%–100% the ascent phase. Across all stance widths, both groups showed similar temporal activation patterns, with relatively low activation during descent, elevated activation during holding, and reduced activation during ascent. Among the three phases, the holding phase was when most muscles exhibited their highest activation levels. In general, the hip-related pain group tended to show greater normalized EMG amplitudes than the control group in several hip and thigh muscles, with the between-group differences most evident during the holding phase. This trend was observed across the 40-, 60-, and 80-cm stance widths, suggesting that stance width did not markedly alter the timing of muscle activation, although it may have influenced activation magnitude. Increased activation in the hip-related pain group was more apparent in muscles related to hip stabilization and lower-limb support, whereas some muscles showed substantial overlap between groups. In both groups, the general shape of the activation curves remained relatively consistent across stance widths, indicating limited influence of stance width on the overall activation sequence. However, the extent of between-group separation varied by stance width, implying that different stances may alter muscular demand in specific muscles.

**Figure 4:**
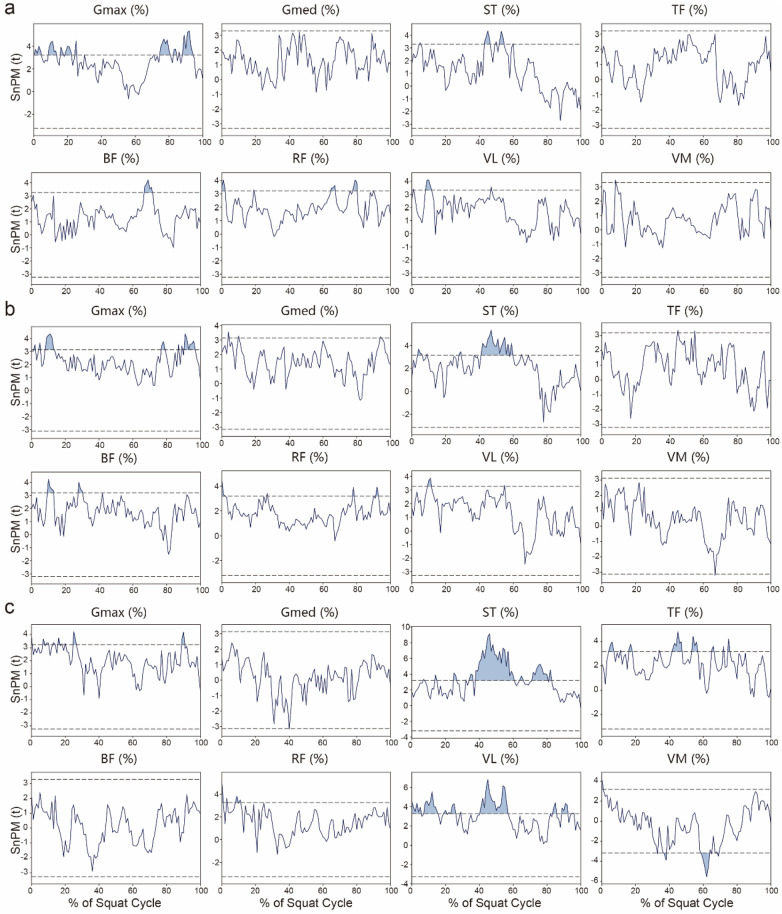
SnPM(t) of EMG signals from eight muscles during squats. **(a)** SnPM(t) trajectories over the full squat cycle (0%–100%) comparing the control and hip-related pain groups during squats with a 40-cm inter-foot distance. **(b)** Corresponding SnPM(t) trajectories for the 60-cm stance. **(c)** Corresponding SnPM(t) trajectories for the 80-cm stance. Each panel shows one muscle, with the *x*-axis representing % of the squat cycle and the *y*-axis the SnPM(t) statistic. Dashed horizontal lines indicate the supra-threshold critical values; shaded regions denote time intervals where SnPM(t) exceeds the threshold, suggesting significant between-group differences in muscle activation during those phases of the squat (*p* < 0.05).

To further assess time-varying between-group differences across the full squat cycle, the normalized EMG waveforms were compared using statistical parametric mapping for time series (SnPM(t)) (Figures 4a–c). To validate the inference of SnPM, the FWHM of the residual field was calculated (Table 2). At the same time, the results showed that the FWHM values for all muscles across different stance-width conditions were approximately 3, indicating similar smoothness. Between-group differences were stance-width dependent. At 40 cm, significant clusters were observed mainly for Gmax during the latter portion of the cycle (primarily within ascent), with smaller significant segments present for VL, ST, and BF; the remaining muscles showed no significant clusters. At 60 cm, significant differences were dominated by ST, which exhibited a continuous significant cluster during the hold phase, with smaller significant segments also observed for Gmax, BF, and VL; other muscles showed no significant differences. At 80 cm, both the number of muscles and the temporal extent of significant differences increased: ST showed a broader significant cluster during the hold phase and an additional significant segment in early ascent; VL showed significant segments in early descent and mid-hold; and VM showed a significant segment in late hold. Overall, SnPM(t) results indicated that between-group differences were primarily concentrated in the hold phase and the transition into ascent, and became more prominent and involved more muscles as stance width increased.

**Table 2:** FWHM values of muscle activation for different stance widths.

Muscle	40 cm	60 cm	80 cm
BF	3.50 (3.5%)	3.39 (3.4%)	3.57 (3.6%)
ST	2.75 (2.7%)	2.85 (2.8%)	2.05 (2.1%)
VL	3.34 (3.3%)	3.40 (3.4%)	3.44 (3.4%)
RF	3.28 (3.3%)	3.29 (3.3%)	2.89 (2.9%)
VM	3.04 (3.0%)	2.99 (3.0%)	2.69 (2.7%)
TF	3.14 (3.1%)	2.80 (2.8%)	2.66 (2.7%)
GMED	2.58 (2.6%)	2.75 (2.7%)	2.74 (2.7%)
GMAX	3.11 (3.1%)	3.16 (3.2%)	2.84 (2.8%)

### Muscle synergy organization and activation timing during squatting

**Figure 5:**
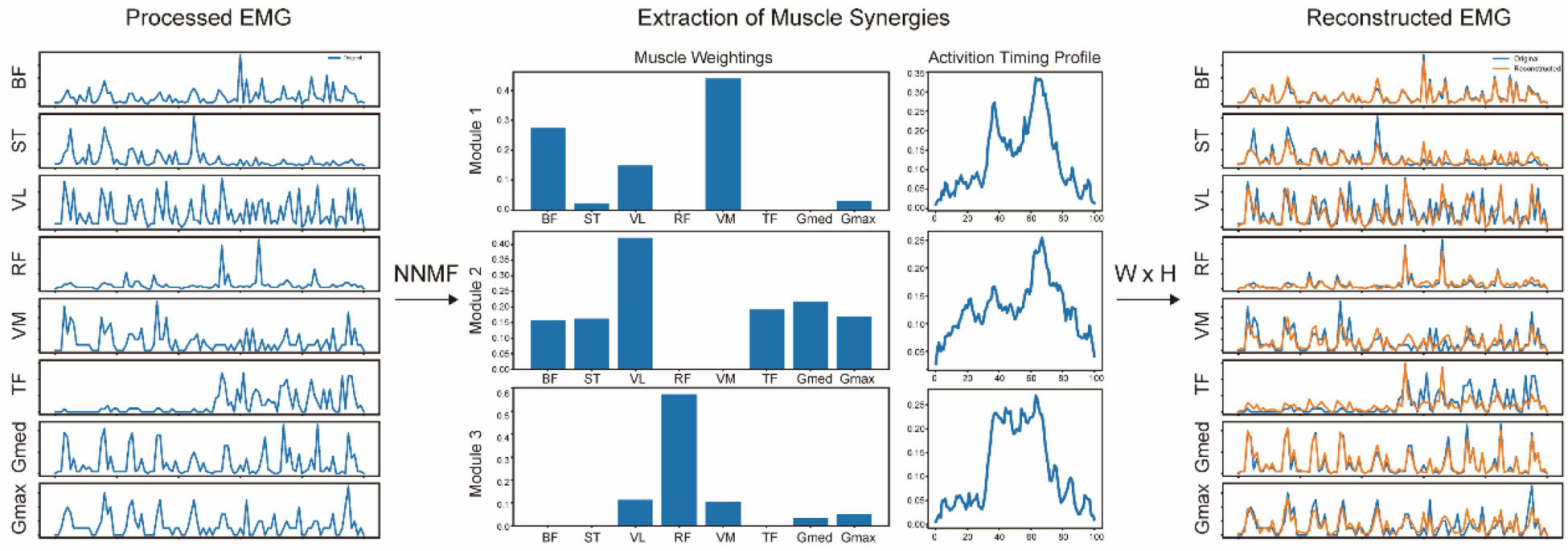
Muscle synergy extraction and EMG reconstruction using non-negative matrix factorization. Left: pre-processed, normalized EMG time series from eight muscles (BF, ST, VL, RF, VM, TF, Gmed, Gmax). Middle: NNMF decomposes the EMG data matrix into a muscle weighting matrix W and an activation timing matrix H across the gait cycle; each synergy/module (Module 1–3) contributes to a muscle's activation as W × H. Right: reconstructed EMG obtained from W × H overlaid with the original EMG; the variance accounted for VAF is computed from their differences to quantify reconstruction accuracy.

**Figure 6:**
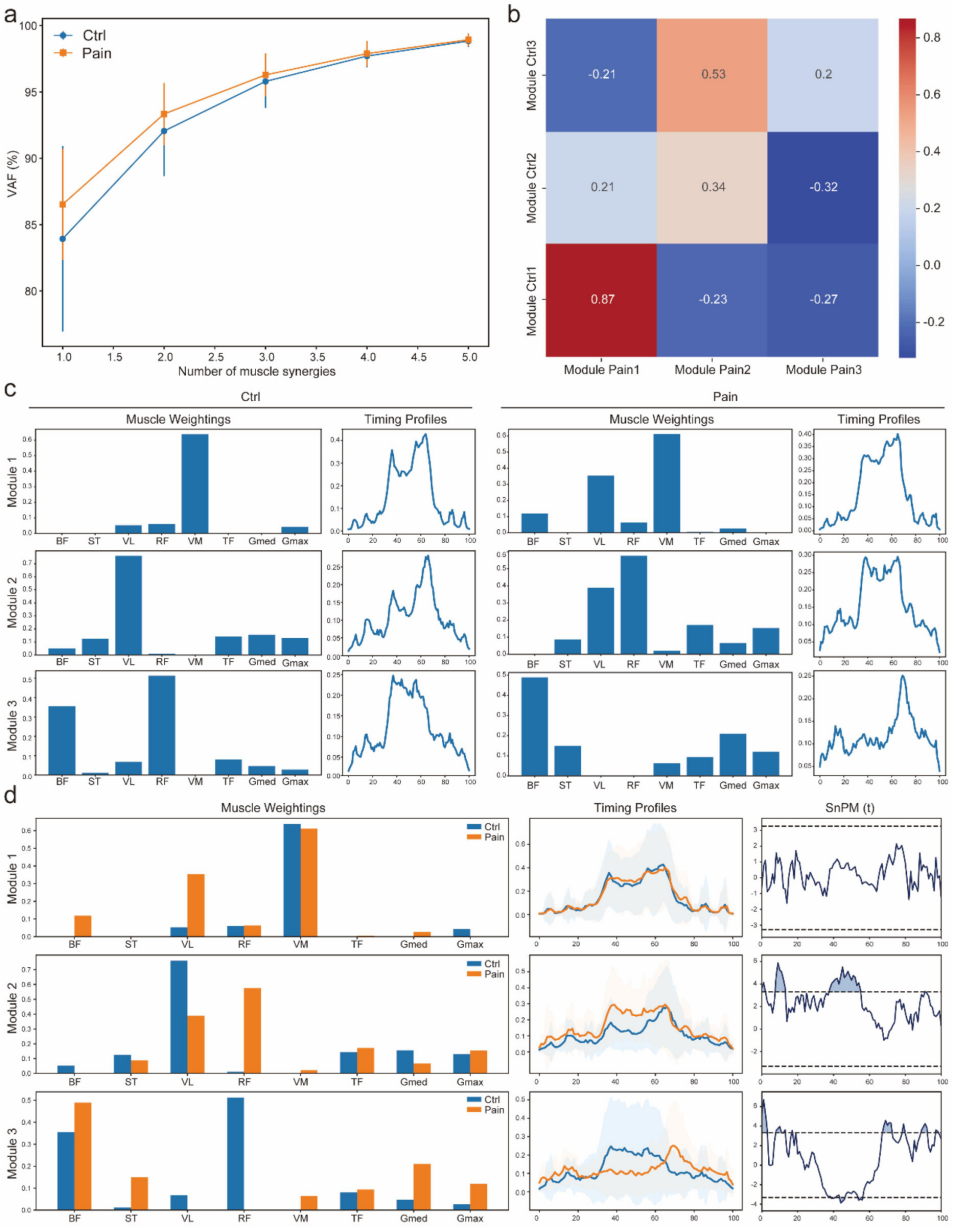
Muscle synergy analysis during 40-cm stance-width squats in the control and hip-related pain groups. **(a)** Group mean VAF (%) of reconstructed EMG as a function of the number of extracted synergies. Symbols indicate group means and error bars indicate s.d. **(b)** Correlation coefficients between synergy muscle weightings from the control groups and the pain groups. Warmer colors indicate higher positive correlations and cooler colors indicate lower or negative correlations; values are shown within each cell. **(c)** Module muscle weightings and activation timing profiles for each synergy in controls and in the pain group. For each module, bar plots indicate the relative weighting of each muscle, and the adjacent line plot shows the corresponding activation coefficient over the normalized squat cycle (0%–100%). **(d)** Between-group comparisons of module structure and activation. Left: muscle weightings for each module in the control (blue) and pain (orange) groups. Middle: group mean activation timing profiles over the squat cycle with shaded regions indicating between-subject variability. Right: statistical parametric mapping SnPM results for the group difference in timing profiles across the cycle (SnPM(t)); dashed horizontal lines indicate the critical threshold for significance.

We first applied non-negative matrix factorization to each participant's EMG data to extract muscle synergy patterns (Figure 5) and determined the minimum number of patterns required to achieve VAF > 90% (Figure 6a). The squat cycle was time-normalized to 0%–100%, with 0%–22% defined as the descent phase, 22%–78% as the hold phase, and 78%–100% as the ascent phase. For the 40-cm stance width, when the number of patterns was set to three, all participants achieved VAF > 90%; therefore, three patterns were retained for subsequent analyses at both the individual and group levels and are hereafter referred to as Modules 1–3, each characterized by a muscle-weighting vector (W) and an activation timing profile (H).

To identify cross-group module correspondence, correlations between W vectors from the control and hip-pain groups were computed and visualized as a heatmap (Figure 6b). For the 40-cm stance width, the strongest correspondence was observed for Ctrl 1—Pain 1 (*r* = 0.87), suggesting high similarity in muscle-weighting structure between groups for Module 1. notably, Pain 3 did not show a clear high-correlation match with any control module, suggesting a more distinct weighting pattern in the hip-pain group. In the control group, Module 1 was dominated by the VM and increased from late descent, with peaks around the end of descent/near the bottom position and again during early-to-mid ascent. Module 2 was dominated by the VL, with additional contributions from the ST, TF, gluteus medius (Gmed), and Gmax; it reached its maximum during ascent and exhibited a lower amplitude than Module 1. Module 3 was characterized by co-activation of the RF and BF and showed a sustained activation plateau predominantly during the hold phase. In the hip-pain group, Module 1 was primarily weighted on VM and VL, with a relatively stronger BF contribution, and remained elevated from the bottom region through early ascent. Module 2 was primarily weighted on VL and RF, with additional weighting from TFL, Gmed, Gmax, and ST, and demonstrated a prolonged activation plateau from the hold phase into early ascent. Module 3 was BF-dominant with increased contributions from ST and the gluteal muscles (Gmed and Gmax) and showed a more pronounced peak during late ascent (Figure 6c).

Between-group differences in W and H were evaluated across the full squat cycle using SnPM(t) (Figure 6d). The FWHM values for all muscles across different stance-width conditions were approximately 3, indicating sufficient smoothness for the analysis (Table 3). At 40 cm, Module 1 was VM-dominant in both groups with comparable timing profiles, and SnPM(t) did not exceed the critical threshold across the cycle. Module 2 demonstrated clearer between-group differences: the control group was VL-dominant, whereas the hip-pain group showed increased RF weighting, relatively reduced VL weighting, and continued involvement of hip-related muscles; temporally, the hip-pain group exhibited a higher and more sustained plateau during the mid-cycle portion, with SnPM(t) exceeding the threshold in this interval. Module 3 exhibited phase-dependent differences: the control group showed RF–BF co-contribution, whereas the hip-pain group was BF-dominant with increased ST and gluteal weighting; temporally, the control group showed higher activation during the hold phase (22%–78%), while the hip-pain group displayed a more pronounced peak during late ascent (78%–100%). Consistently, SnPM(t) revealed a significant negative cluster during the hold phase and a significant positive cluster during late ascent. Overall, between-group differences at 40 cm were primarily concentrated in Modules 2 and 3, reflecting differences in both W structure and phase-dependent timing modulation.

**Table 3:** FWHM values for muscle synergies across different stance widths.

Muscle	40 cm	60 cm	80 cm
Syn1	3.12 (3.1%)	3.08 (3.1%)	2.74 (2.7%)
Syn2	3.45 (3.5%)	3.05 (3.1%)	2.41 (2.4%)
Syn3	3.92 (3.9%)	3.44 (3.4%)	3.30 (3.3%)

To further quantify cross-group similarity, a cross-reconstruction analysis was performed at 40 cm, in which group-derived module vectors were used to reconstruct EMG from both groups and muscle-wise VAF changes were computed (Table 4). The total VAF change did not differ significantly between groups (*p* > 0.05). However, significant muscle-specific differences were observed for RF and Gmed (*p* < 0.05), suggesting that between-group differences were localized to specific module components rather than reflecting a global shift in module structure.

**Table 4:** Change in VAF for each muscle when EMG patterns are reconstructed with the pain-group vector and the control-group vector during squats at 40-cm stance width.

Muscle	Pain Group Vector			Control Group Vector		
	Pain	Control	*p*-value	Pain	Control	*p*-value
Total	−6.65 ± 3.96	−12.72 ± 7.28	0.9832	−10.57 ± 5.51	−8.77 ± 4.11	>0.9999
BF	−2.58 ± 11.29	−5.98 ± 16.60	0.9998	−28.75 ± 17.76	−20.61 ± 8.12	0.9168
ST	−27.08 ± 28.29	−29.51 ± 26.32	>0.9999	−35.64 ± 22.15	−37.41 ± 33.14	>0.9999
VL	−3.79 ± 5.86	−5.98 ± 5.67	>0.9999	1.61 ± 4.29	3.52 ± 7.86	>0.9999
RF	−3.21 ± 4.13	−30.86 ± 42.80	0.0004	−8.56 ± 14.32	−6.25 ± 10.33	>0.9999
VM	−2.03 ± 5.17	1.09 ± 2.42	0.9999	2.26 ± 2.84	2.24 ± 3.00	>0.9999
TF	−33.22 ± 29.41	−24.95 ± 18.87	0.8889	−34.92 ± 21.51	−35.86 ± 28.89	>0.9999
GMED	−20.63 ± 17.22	−20.56 ± 17.13	>0.9999	−35.02 ± 25.14	−55.13 ± 32.05	0.0355
GMAX	−29.41 ± 20.92	−19.16 ± 7.22	0.7037	−30.12 ± 23.31	−23.48 ± 13.87	0.9757

**Figure 7:**
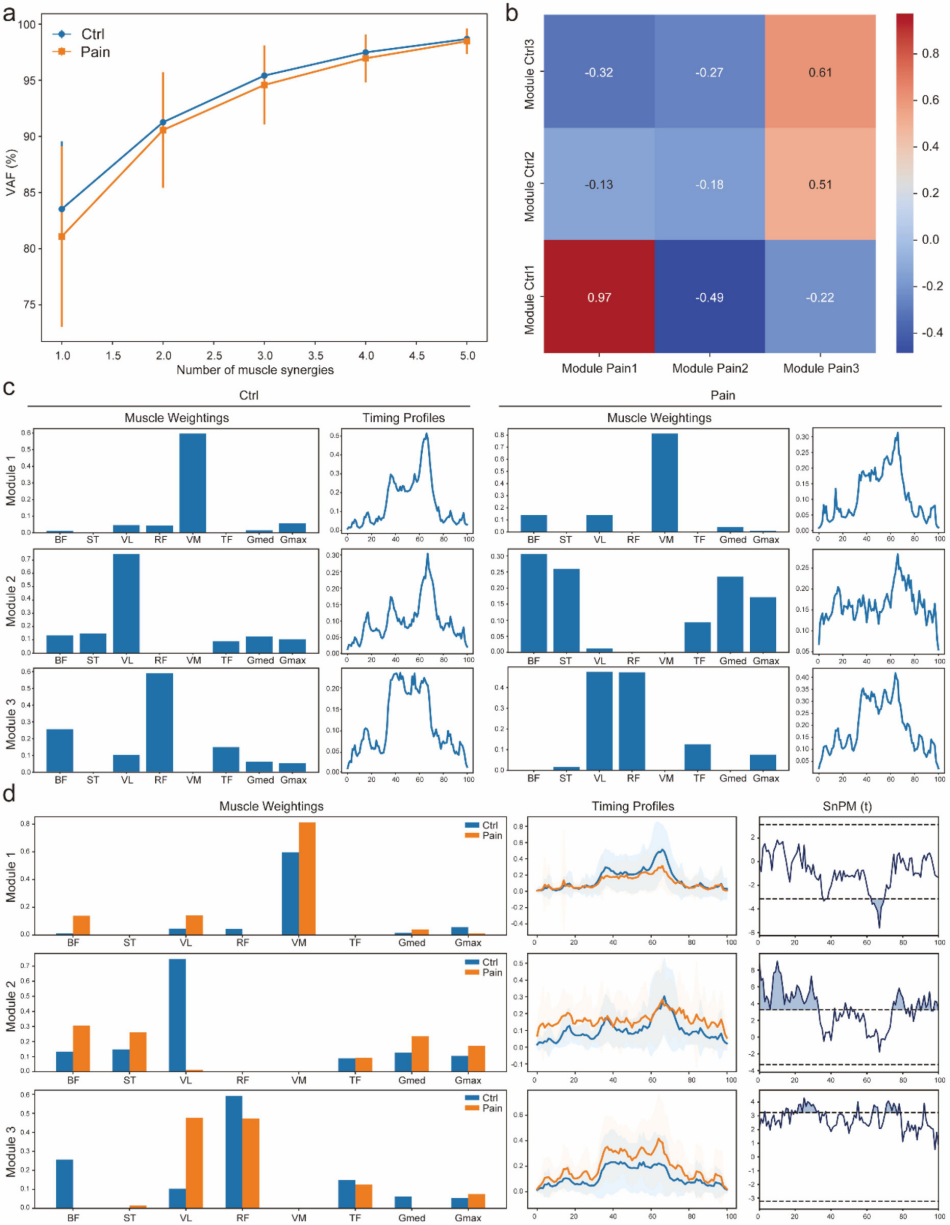
Muscle synergy analysis during 60-cm stance-width squats in the control and hip-related pain groups. **(a)** Group mean VAF (%) of reconstructed EMG as a function of the number of extracted synergies. Symbols indicate group means and error bars indicate s.d. **(b)** Correlation coefficients between synergy muscle weightings from the control groups and the pain groups. Warmer colors indicate higher positive correlations and cooler colors indicate lower or negative correlations; values are shown within each cell. **(c)** Module muscle weightings and activation timing profiles for each synergy in controls and in the pain group. For each module, bar plots indicate the relative weighting of each muscle, and the adjacent line plot shows the corresponding activation coefficient over the normalized squat cycle (0%–100%). **(d)** Between-group comparisons of module structure and activation. Left: muscle weightings for each module in the control (blue) and pain (orange) groups. Middle: group mean activation timing profiles over the squat cycle with shaded regions indicating between-subject variability. Right: statistical parametric mapping SnPM results for the group difference in timing profiles across the cycle (SnPM(t)); dashed horizontal lines indicate the critical threshold for significance.

Building on the 40-cm findings, increasing stance width to 60 cm yielded a similar VAF–module relationship, with both groups exceeding VAF > 90% when three modules were extracted (Figure 7a); therefore, three modules were likewise retained for analysis at 60 cm. The correlation heatmap (Figure 7b) indicated that only Module 1 showed a clear cross-group correspondence, whereas the remaining modules exhibited weak or inconsistent correlations, making one-to-one matching difficult at 60 cm. Regarding module composition and timing, the control-group modules were broadly similar to those observed at 40 cm. In contrast, the hip-pain group showed a more pronounced redistribution, with Module 1 remaining VM-dominant but with increased BF/VL contribution, Module 2 becoming BF/ST-dominant with additional hip-muscle involvement, and Module 3 primarily weighted on VL and RF (Figure 7c). Time-series testing of the activation profiles (H) using SnPM(t) revealed phase-dependent differences: Module 1 showed an overall similar waveform but exhibited a significant cluster during the hold phase; Modules 2 and 3 demonstrated more extensive differences across the squat cycle, with significant clusters mainly during descent and hold, extending into parts of ascent (Figure 7d). Overall, at 60 cm both groups were still well represented by three modules, but between-group differences and module matching were more prominent for Modules 2 and 3, particularly around descent–ascent transition-related portions of the task. The total VAF change did not differ significantly between groups (*p* > 0.05), suggesting overall similarity at the aggregate level; however, a significant muscle-specific difference was observed for ST (Table 5).

**Table 5:** Change in VAF for each muscle when EMG patterns are reconstructed with the pain-group vector and the control-group vector during squats at 60-cm stance width.

Muscle	Pain Grouph Vector			Control Group Vector		
	Pain	Control	*p*-value	Pain	Control	*p*-value
Total	−12.12 ± 8.64	−11.09 ± 5.52	>0.9999	−16.73 ± 14.42	−8.32 ± 3.81	0.9363
BF	−14.50 ± 18.09	−8.48 ± 8.65	0.9736	−27.43 ± 14.16	−27.67 ± 17.24	>0.9999
ST	−14.71 ± 13.63	−17.42 ± 24.58	>0.9999	−52.40 ± 26.28	−29.22 ± 30.34	0.0201
VL	−7.67 ± 22.02	−9.92 ± 6.24	>0.9999	−3.48 ± 8.59	0.75 ± 13.44	0.9995
RF	−11.48 ± 15.41	−16.51 ± 12.45	0.9923	−2.56 ± 11.55	2.21 ± 11.74	0.9988
VM	5.06 ± 9.70	2.19 ± 3.58	>0.9999	5.93 ± 9.66	2.69 ± 3.92	>0.9999
TF	−25.53 ± 23.20	−38.46 ± 27.26	0.2940	−26.27 ± 25.16	−52.08 ± 27.51	0.0062
GMED	−21.17 ± 15.52	−22.98 ± 18.45	>0.9999	−43.42 ± 26.29	−43.35 ± 29.49	>0.9999
GMAX	−20.40 ± 11.70	−29.44 ± 19.78	0.7581	−38.09 ± 25.25	−29.80 ± 20.53	0.9414

**Figure 8:**
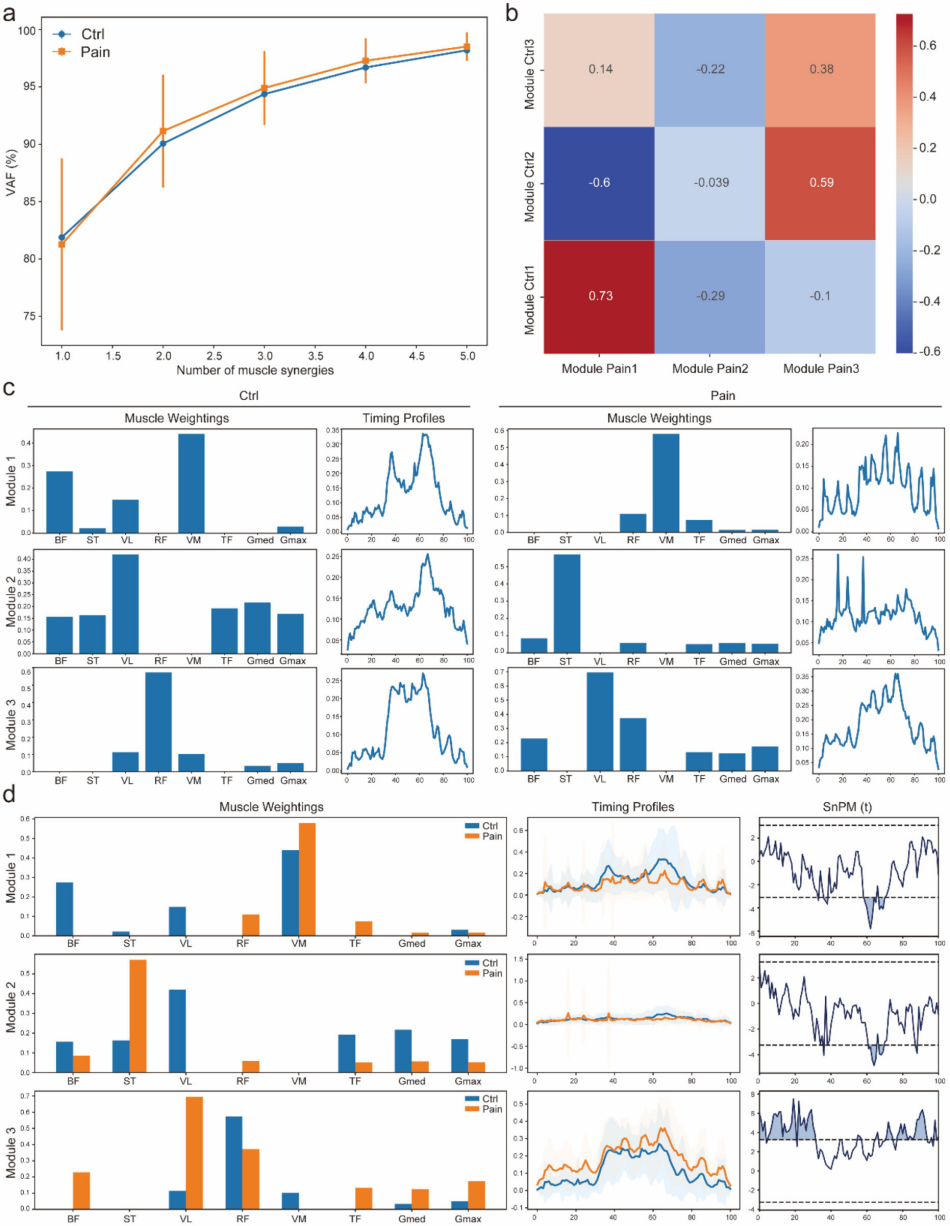
Muscle synergy analysis during 80-cm stance-width squats in the control and hip-related pain groups. **(a)** Group mean VAF (%) of reconstructed EMG as a function of the number of extracted synergies. Symbols indicate group means and error bars indicate s.d. **(b)** Correlation coefficients between synergy muscle weightings from the control groups and the pain groups. Warmer colors indicate higher positive correlations and cooler colors indicate lower or negative correlations; values are shown within each cell. **(c)** Module muscle weightings and activation timing profiles for each synergy in controls and in the pain group. For each module, bar plots indicate the relative weighting of each muscle, and the adjacent line plot shows the corresponding activation coefficient over the normalized squat cycle (0%–100%). **(d)** Between-group comparisons of module structure and activation. Left: muscle weightings for each module in the control (blue) and pain (orange) groups. Middle: group mean activation timing profiles over the squat cycle with shaded regions indicating between-subject variability. Right: statistical parametric mapping SnPM results for the group difference in timing profiles across the cycle (SnPM(t)); dashed horizontal lines indicate the critical threshold for significance.

When stance width was further increased to 80 cm, the VAF criterion again supported extraction of three modules, with both groups achieving VAF > 90% (Figure 8a); thus, three modules were retained for comparison at 80 cm. The correlation heatmap (Figure 8b) showed the highest correspondence for Ctrl 1–Pain 1 (*r* = 0.73), whereas correlations among the remaining modules were generally low (near zero or negative), suggesting unclear cross-group module correspondence beyond Module. Module composition and timing suggested a stance-dependent shift in the control group relative to narrower stances: Module 1 remained VM-dominant with BF and VL involvement; Module 2 remained VL-dominant with contributions from ST, TF, Gmed, and Gmax but with smaller relative weights than at 40 and 60 cm; and Module 3 was RF-dominant. In the hip-pain group, Module 1 remained VM-dominant with additional RF and TF involvement; Module 2 was primarily ST-dominant; and Module 3 was dominated by VL and RF with additional posterior-chain and hip-muscle contributions (Figure 8c). SnPM(t) testing of H indicated that Module 1 differed from late hold into early ascent, Module 2 showed phase-specific differences during ascent, and Module 3 exhibited significant clusters spanning large portions of descent and ascent (Figure 8d). Overall, at 80 cm both groups were still represented by three modules, but between-group differences remained concentrated in Modules 2 and 3, with a broader temporal extent than at 60 cm. The total VAF change again did not differ significantly between groups (*p* > 0.05), suggesting similarity in overall reconstruction performance at the aggregate level (Table 6).

**Table 6:** Change in VAF for each muscle when EMG patterns are reconstructed with the pain-group vector and the control-group vector during squats at 80-cm stance width.

Muscle	Pain Group Vector			Control Group Vector		
	Pain	Control	*p*-value	Pain	Control	*p*-value
Total	−14.17 ± 9.73	−14.03 ± 8.04	>0.9999	−17.46 ± 9.54	−8.82 ± 4.54	0.7516
BF	−26.00 ± 19.20	−29.99 ± 17.45	0.9988	−19.92 ± 17.77	−43.67 ± 57.66	0.5588
ST	8.58 ± 9.28	15.41 ± 8.45	0.9476	−36.92 ± 19.47	−28.40 ± 16.12	0.7654
VL	−6.32 ± 13.47	−6.37 ± 7.54	>0.9999	−11.19 ± 11.43	−7.66 ± 11.85	0.9992
RF	−17.04 ± 13.36	−24.60 ± 17.26	0.9062	7.40 ± 10.69	3.58 ± 6.73	0.9986
VM	5.40 ± 9.36	2.19 ± 2.08	0.9998	−2.77 ± 9.41	−1.17 ± 3.19	>0.9999
TF	−33.11 ± 19.53	−31.46 ± 34.38	>0.9999	−34.78 ± 20.39	−18.76 ± 24.63	0.0609
GMED	−40.24 ± 19.15	−22.71 ± 24.11	0.0509	−28.91 ± 17.87	−12.83 ± 18.01	0.0592
GMAX	−27.26 ± 17.75	−22.73 ± 25.62	0.9969	−24.85 ± 19.06	−17.30 ± 19.85	0.8668

## Discussion

The present study demonstrates that individuals with early-stage, non-surgical chronic hip pain rely on a task- and phase-dependent reorganization of neuromuscular strategies during squatting, despite preserved overall movement patterns. A central finding is the distinct dissociation between maximal force capacity and functional muscle demand: the hip-pain group displayed globally reduced MVICs (Figure 3)—indicating potential arthrogenic muscle inhibition or early disuse—yet demonstrated significantly higher relative activation (%MVC) (Figure 2) and cumulative recruitment (iEMG) (Figure 3) during the squat task. This elevated activation was most pronounced during the isometric holding phase (Figure 1**)**. However, the temporal activation profiles across the squat cycle remained remarkably consistent between groups, with no significant shifts in activation timing (Figure 1). Taken together, these results indicate that the central nervous system preserves the basic movement pattern rather than modifying it, but instead increases neural drive and sends enhanced control signals to the musculature to maintain task performance. Consequently, these individuals can successfully execute the task but operate in a state of neuromuscular inefficiency, requiring a higher physiological cost that may accelerate local muscle fatigue during daily activities.

This compensatory upregulation was not uniform, but rather heavily favored the knee extensor mechanism and specific hip stabilizers. The robust between-group differences observed in the quadriceps (e.g., RF, VL, VM) compared to primary hip extensors (Figures 2b, 3) suggest a protective load-shifting strategy, where patients instinctively adopt knee-dominant mechanics to offload the symptomatic hip joint. Furthermore, varying the stance width provided additional biomechanical insights. While widening the stance generally amplified integrated EMG in most muscles, it uniquely reduced vastus medialis recruitment (Figure 3). This divergent VM response likely reflects a mechanical trade-off at wider stances—such as altered adduction or internal rotation moments—that specifically modifies localized stabilization requirements. Collectively, these findings confirm that early hip-related pain is not characterized by a global breakdown in motor control, but rather drives targeted, strategy-level adaptations to accommodate underlying neuromuscular deficits and protect the affected joint.

Previous biomechanical studies have demonstrated that stance width influences lower-limb joint loading patterns and increases frontal-plane stability demands during squatting (23, 24). However, whether stance width can systematically reveal early neuromuscular control differences in individuals with hip-related pain, particularly when overt functional performance is preserved, has remained unclear. In the present study, between-group differences were relatively limited at the narrowest stance width, but as stance width increased, the differences in both the magnitude of muscle activation and the timing of activation became progressively more pronounced (Figures 6–8): at wider stances, group differences expanded both in temporal extent and in the number of muscles involved, with differences emerging most clearly in modules associated with multi-joint coordination and stability control. At wider stance widths, between-group differences not only expanded in temporal duration but also involved a greater number of muscles, particularly within synergy modules associated with multi-joint coordination and stability control. As shown in Figures 8c,d, at the 80 cm stance width, individuals with hip pain exhibited markedly reduced rectus femoris force during both the descending and ascending phases of the squat compared with controls. This deficit was accompanied by more widespread activation of the biceps femoris, vastus lateralis, tensor fasciae latae, gluteus medius, and gluteus maximus, suggesting a compensatory redistribution of muscular effort. At the 60 cm stance width (Figures 7c,d), during both the descent and ascent phases, the activation strategy in the hip pain group shifted from being primarily driven by the vastus lateralis to a more coordinated pattern involving the semitendinosus, biceps femoris, and gluteal muscles. In contrast, at the narrowest stance width of 40 cm, SnPM analysis indicated that between-group differences during the squat cycle were substantially less pronounced than those observed at the wider stance conditions (Figures 6c,d). These findings suggest that stance width functions not merely as a task configuration parameter, but as a meaningful constraint that limits available motor solutions. As stability demands increase, individuals with hip-related pain appear less able to rely on flexible movement strategies to mask underlying control alterations, thereby exposing compensatory neuromuscular patterns that remain concealed under lower-demand conditions. From this perspective, increasing stance width acts as an effective “amplifier” of latent neuromuscular control differences in early-stage hip-related pain.

Previous studies in populations with more advanced pathology or following surgical intervention have reported marked alterations in muscle synergy structure, including reductions in the number of synergies or degradation of modular organization, which are commonly interpreted as reflecting impaired or simplified neuromuscular control (25–27). In contrast, the present study demonstrated that individuals with early-stage or non-surgical hip-related pain preserved the same number of muscle synergies as asymptomatic controls across all stance-width conditions, with comparable overall reconstruction quality (Figures 6–8a,b). These findings suggest that the fundamental dimensionality of neuromuscular control remains intact at this stage. However, between-group differences were not expressed as a loss of synergy number or global modular capacity, but rather as reorganization of specific synergies in terms of weighting and activation timing. Further analyses indicated that this reorganization was functionally selective: support-related activation dominated by the quadriceps remained relatively conserved across stance-width conditions, whereas under wider stance conditions, differences in activation magnitude and timing involving the hamstrings, hip abductors, and associated synergistic muscle groups were markedly amplified during the hold phase and the transition from holding to rising (Figures 6–8c,d). This pattern indicates that, in early-stage hip-related pain, the neuromuscular system does not undergo global degradation, but instead prioritizes the preservation of task-essential support strategies while reorganizing stability and coordination-related synergies to accommodate increased control demands.

Time-series analyses further revealed that this strategy-level reorganization did not occur uniformly across the squat cycle, but was primarily concentrated during the hold phase and the transition from holding to rising, with this phase specificity becoming more pronounced as stance width increased. These phases correspond to periods of the squat that impose the greatest demands on sustained force production and postural stability (Figures 6–8c,d). During the hold phase, maintaining a quasi-static posture requires continuous neuromuscular regulation to counteract gravitational and mediolateral perturbations, whereas the transition to rising requires rapid redistribution of joint moments and coordinated activation across multiple muscle groups (28–30). In the present study, individuals with hip-related pain exhibited higher and more sustained muscle activation, as well as adjustments in synergy activation timing during these phases, suggesting a greater reliance on stabilizing strategies to compensate for reduced control efficiency. This phase-specific synergy reorganization provides a direct neuromuscular explanation for why functional task performance may be preserved in early-stage hip-related pain despite underlying alterations in compensatory neuromuscular control strategies, and further explains why discrete peak or cycle-averaged metrics often fail to capture these early changes.

From a clinical perspective, these findings indicate that the ability to perform normal movements in patients with early hip-related pain should not be conflated with preserved neuromuscular function. Compensatory control strategies may already be established, though they remain masked under conditions of low task demand. Previous literature demonstrates that compensatory motor control strategies are often concealed during low-demand tasks and only become evident when stability or coordination requirements are elevated (31, 32). Our results corroborate this perspective, suggesting that manipulating stance width during functional tasks such as squatting serves as a simple yet effective method to heighten stability demands. Consequently, this positions the stance-manipulated squat as a standardized task for investigating underlying pathological mechanisms. However, it must be acknowledged as a limitation that we did not evaluate whether individuals with FAI syndrome experienced greater pain than healthy controls during stance width manipulation. Identifying an increase in task-specific pain would be a highly clinically relevant finding, potentially establishing this squat variation as a clinically meaningful observational test. Future research should incorporate pain assessments alongside comprehensive biomechanical methodologies to further elucidate potential disparities in neuromuscular control.

Furthermore, these results underscore that clinical assessments should transcend the mere ability to complete a task and instead focus on *how* the movement is executed—a paradigm widely endorsed in functional assessment and motor control research (33, 34). Early identification of an over-reliance on sustained, high-intensity muscle activation patterns may facilitate timely interventions before these compensatory strategies become entrenched or precipitate secondary periarticular tissue overload. Nevertheless, because the gross kinematics of the squat are visually preserved in patients with FAI syndrome compared to healthy individuals, discerning these subtle discrepancies in muscle activation patterns necessitates specialized equipment, such as surface electromyography. Consequently, while our findings contribute significantly to the understanding of latent neuromuscular control deficits in this population, the immediate clinical application of this assessment protocol may be currently limited to clinics equipped with specialized instrumentation. In rehabilitation practice, the progressive manipulation of stance width could serve a dual purpose: acting not only as an assessment tool but also as a graded training strategy to specifically target improvements in coordination efficiency and movement economy alongside strength gains.

Several limitations of the present study should be acknowledged. First, joint contact forces, co-contraction indices, and musculoskeletal modeling were not directly assessed. Therefore, inferences regarding joint load distribution and tissue stress were based on indirect evidence from the literature. Future studies should integrate multimodal biomechanical data to provide a more comprehensive analysis from both kinematic and kinetic perspectives. Second, repeated measurements before and after intervention were not performed; thus, it remains unclear whether the observed synergy reorganization is reversible. Moreover, hip internal/external rotation angle was not strictly controlled during the squat tasks, which may have influenced lower-limb alignment and muscle activation patterns across participants. Furthermore, although a 50 Hz notch filter was applied to reduce residual power-line interference, this preprocessing step may also have attenuated a small portion of the physiological EMG signal and should therefore be considered when interpreting the EMG results. Third, the relatively small sample size may limit the statistical power and generalizability of the findings. This small sample size, combined with potential non-uniform smoothness in sEMG signals, may have influenced the SnPM cluster analysis. As non-uniform smoothness can result in smaller or more fragmented suprathreshold clusters, the observed differences in certain muscles, such as the Semitendinosus, should be interpreted with caution. These patterns might reflect either genuine neuromuscular control strategies or localized artifacts related to signal processing and inter-individual variability. Future research with larger cohorts is needed to verify these findings and ensure higher spatial-temporal robustness of the SnPM results. Finally, we have verified that all signals used for SnPM analysis and plotting strictly correspond to the fully processed linear envelopes to minimize processing-related bias.

## Conclusion

In individuals with early-stage or non-surgical chronic hip pain, pain-induced muscle stiffness and strength deficits lead to increased reliance on sustained muscle activation and adjustments in muscle synergy strategies to maintain movement. The reduction in maximal strength necessitates higher relative activation to compensate, especially during tasks like squatting, where the hip pain group showed lower maximal voluntary contraction across multiple muscles, suggesting diminished neuromuscular capacity. To adapt to this reduction, individuals with hip pain reorganize their muscle synergy strategies. Muscle synergy analysis revealed preserved overall synergy reconstruction with three modules, but in wider stance conditions, selective reweighting and phase-specific timing adjustments were evident, particularly during the hold phase and the transition to rising. These adjustments reflect the need to meet increased stability and coordination demands in more challenging conditions. Time-varying EMG differences were concentrated in the hold phase and transition to rising, with wider stances involving more muscles. Frequency-domain analysis showed stable indices, suggesting that the observed differences are due to coordination strategy and capacity constraints, not fatigue. In conclusion, the reduction in maximal strength forces individuals with hip pain to use more sustained activation and adapt their muscle coordination. Stance width can serve as an effective tool to reveal these changes and guide rehabilitation, making it a practical parameter for assessing and progressing early hip pain rehabilitation.

## Data Availability

The raw data supporting the conclusions of this article will be made available by the authors, without undue reservation.
